# Academic outcomes following adolescent sport-related concussion or fracture injury: A prospective cohort study

**DOI:** 10.1371/journal.pone.0215900

**Published:** 2019-04-25

**Authors:** Kelly Russell, Erin Selci, Brian Black, Karis Cochrane, Michael Ellis

**Affiliations:** 1 Department of Pediatrics and Child Health, University of Manitoba, Winnipeg, Manitoba, Canada; 2 Children's Hospital Research Institute of Manitoba, Winnipeg, Manitoba, Canada; 3 Canada North Concussion Network, Winnipeg, Manitoba, Canada; 4 Department of Surgery, Section of Neurosurgery, University of Manitoba, Winnipeg, Manitoba, Canada; 5 Pan Am Concussion Program, Winnipeg, Manitoba, Canada; Ohio State University, UNITED STATES

## Abstract

**Objectives:**

The objectives were 1) to compare the effects of adolescent sport-related concussion (SRC) and sport-related extremity fracture (SRF) on academic outcomes including change in school grades and school attendance; and 2) to determine which specific academic accommodations were most helpful during recovery from these injuries.

**Methods:**

A prospective cohort study was conducted to compare changes in school grades, school attendance and academic accommodations among students (grades 8–12) with an SRC or SRF. School grades were extracted from student immediate pre- and post-injury report cards. Students completed attendance log books and an exit interview to determine which accommodations were helpful and how accommodating they perceived their school to be during their recovery.

**Results:**

Overall, 124 students (92 with SRC and 32 with SRF) submitted both pre- and post-injury report cards. Students who sustained an SRC or SRF experienced similar decreases in grades post-injury (SRC: -1.0%; 95% CI: -2.1, 0.1 and SRF: -0.9%: 95% CI: -2.1, 0.3). Students with an SRC missed significantly more days of school compared to those with an SRF (median of 4 days [IQR: 1, 7] versus 1 day [IQR: 0, 4], p<0.0001). In total, 60/113 (53.1%) SRC students reported their school to be very accommodating while only 31/77 (40.3%) SRF students reported their school to be very accommodating (p = 0.082).

**Conclusions:**

Students who sustain an SRC miss significantly more days of school but demonstrate similar changes in school grades post-injury compared to those with an SRF. Future studies are needed to identify the pre- and post-injury factors associated with poor academic functioning following concussion and identify measures that can be taken to help optimize academic outcomes in these patients.

## Introduction

Concussion is the most common form of traumatic brain injury (TBI) and is associated with a rapid onset of neurological symptoms that typically resolve spontaneously [1]. Children and adolescents often experience symptomatic recovery within weeks post-injury; however, approximately 30% develop persistent post-concussion symptoms lasting longer than 1 month [2,3]. Persistent post-concussion symptoms can manifest as headaches, dizziness, visual disturbance, or difficulties with memory and concentration that can have a negative impact on patient functioning and health-related quality of life [4–6]. These symptoms can also negatively impact school performance.

Recently, there has been increased attention on the effects of concussion on school functioning. In one study, 38% of high school student athletes reported difficulties with school following concussion [7]. Returning to school can be associated with increased concussion symptoms [8]. Also, students who return to school with ongoing symptoms reported greater concerns about the impact of their concussion on school performance and more school-related problems that those who have achieved symptomatic recovery [9]. To address the unique challenges of student-athletes with concussion, the 5^th^ International Conference on Concussion in Sport introduced a 4-step Return-to-School Strategy that includes general recommendations allowing for patients make a gradual return to cognitive and school activities [1]. In Canada, the Canadian Pediatric Society has released several Return to Learn recommendations, such as a gradual return to school with increasing time in the classroom providing that symptoms do not worsen, extra time to write exams, and not making up missed assignments and tests [10].

Despite these important advances, few studies have examined the impact of adolescent sport-related concussion (SRC) on objective measures of academic performance. A recent systematic review of students who sustained a concussion or mild TBI found no significant difference in national exam scores or grades but students with a concussion missed more school than those with a non-head injury or no injury [4]. This review included only nine studies that were too heterogeneous to conduct a meta-analysis and no study compared the effects of adolescent SRC to sport-related non-head injuries on school grades and attendance or examined which specific academic accommodations were most helpful during recovery from these injuries.

Therefore, the objectives of this study were 1) to compare the effects of adolescent SRC and sport-related extremity fracture (SRF) on academic outcomes including change in school grades and school attendance; and 2) to determine which specific academic accommodations were most helpful during recovery from these injuries.

## Materials and methods

### Study design and setting

A prospective cohort study was conducted where adolescent students with a diagnosis of SRC were compared to adolescent students with a sport-related upper or lower extremity fracture and no head injury. The comparison group was chosen to account for the effects of non brain-related injury on school functioning and academic outcomes. Orthopedically injured patients have also been used as a non brain-injured control group in previous studies of the effects of concussion or mild TBI in youth [11,12].

This study was conducted at the Pan Am Clinic in Winnipeg, Manitoba. The Pan Am Clinic includes a Minor Injury for Kids Clinic and the Pan Am Concussion Program. The Pan Am Concussion Program is a provincial pediatric concussion program that receives referrals from primary care providers and emergency departments throughout the province of Manitoba. The Minor Injury for Kids Clinic provides acute and follow-up care for pediatric patients who sustain orthopaedic injuries, including extremity fractures, and receives referrals from outside primary care physicians and emergency departments in Manitoba.

### Inclusion criteria

#### Sport-related concussion

Students who were enrolled in a Winnipeg high school (grades 8–12) and were diagnosed with an SRC within one month of the date of injury were included. The diagnosis of acute SRC was made by a single neurosurgeon according to the International Concussion in Sport Group Consensus Statement [1]. Sport-related concussion was diagnosed in patients who sustained a traumatic impulsive force to the head or body during a sporting activity and presented with new concussion-like symptoms such as headaches, dizziness, fatigue, and difficulty concentrating or remembering that could not be attributed to an alternative medical diagnosis. Students with a history of moderate/severe traumatic brain injury, abnormal neuroimaging findings (e.g., intracranial haemorrhage), those who presented with persistent post-concussion symptoms at the time of initial assessment (symptoms >1 month post-injury), or those with co-existing injuries were excluded.

#### Sport-related fracture

Students who were diagnosed with an acute sports-related fracture involving an upper or lower extremity and were enrolled in a Winnipeg high school (grades 8–12) at the time of injury were included. The diagnosis of SRF was made by a pediatric orthopaedic surgeon or pediatric primary care physician based on the results of clinical and radiographic findings. Students with multiple injuries, those requiring surgical repair of their fracture, or who presented more than 30 days after their injury were excluded. Students were also excluded if they were diagnosed with a co-existing concussion by the physician.

### Participant recruitment

Students with a SRC were recruited from the Pan Am Concussion Program from October 2014 to October 2015. The neurosurgeon informed the student (and their parent) of their eligibility following initial clinical assessment. If the student and parent were interested in participating, they were asked to meet with the Research Assistant (RA) who explained the study and obtained written parental consent and written assent from the student.

Students with a fracture injury were recruited from the Minor Injury for Kids Clinic at Pan Am Clinic. After the initial clinical assessment, the treating physician would alert the RA to a potentially eligible patient and the RA then described the study and sought parental consent and student assent in the same manner as the concussed students.

### Outcome measures: Academic performance, school attendance, academic accommodations

The primary outcome of this study was change in overall grades from pre- and post-injury report cards. Secondary outcomes included change in core subject grades, days missed from school (both full days and specific classes) and perceptions of specific school-related accommodations. Core subjects included English, Sciences, Maths, Social Studies, and Foreign Languages.

### Data collection and follow-up

For students with a SRC, the initial visit consisted of an initial clinical interview, symptom inventory, and physical examination conducted by the neurosurgeon. Additional variables were also collected during the initial visit: age, grade, sex, past medical history, and sport being played at the time of injury. Concussion symptoms were measured using the Post-Concussion Symptom Scale (PCSS), a standardized 22-item scale that allows patients to rate the severity of individual concussion symptoms on a 7-point Likert scale [13,14]. For students with a SRF, the initial visit consisted of a clinical interview, physical examination, and review of diagnostic imaging conducted by a pediatric orthopedic surgeon or primary care physician. With the exception of concussion symptoms, the same additional variables were collected for both groups of students. At the end of their visit, students were given a log book to track their school attendance until their next appointment. Students were instructed to only record school days missed due to their injury.

Patients were seen at follow up at intervals based on the discretion of the treating physician. Based on the nature and severity of presenting concussion symptoms, some SRC patients were provided with an individually-tailored Return-to-Learn program that outlined specific academic accommodations that may benefit the patient while making a return to school. Return-to-Learn programs were provided at the discretion of the treating neurosurgeon and not based on any standardized criteria. At the time of this study, there were no established guidelines to identify which students should be provided with individualized Return-to-Learn programs following pediatric concussion. In general, Return-to-Learn programs were considered for patients who presented with more severe symptoms and who were anticipated to experience a more prolonged recovery or difficulty returning to school. These programs were not considered in patients that presented with less symptoms, who were expected to experience a shorter recovery (e.g. 1–2 weeks) and had made a successful return to school at the time of initial assessment. Return-to-Learn programs included accommodations such as limiting time reading and using computers, allowing students to take breaks if they experienced worsening concussion symptoms, and allowing students extra time to complete tests and assignments. Patients who did not receive an individually-tailored Return-to-Learn program were provided general education on how to make a gradual return to school. In general, all SRC patients were advised by the neurosurgeon to initiate a gradual return to school as soon as they felt like they could tolerate doing so. Parents of both patient groups were requested to bring their child’s most recent pre-injury report card to their subsequent visit(s). At each subsequent visit, students were asked to return their previous log book and were given a new one. Following medical clearance to return to sports or discharge from clinical care, the RA administered a five-minute in-person structured exit interview to assess which academic accommodations were provided by the school and if the student perceived their school to be accommodating. Students were presented with a list of accommodations and asked to rate each as very helpful, somewhat helpful, not helpful, not available, or not applicable. Students were not asked to rate the accommodations but to consider the helpfulness of each accommodation. Finally, the RA telephoned parents to ask them for their child’s first report card issued immediately after they recovered from their injury.

Length of physician-documented clinical recovery was defined as the number of days from the date of injury until physician-documented clinical recovery. In general, SRC patients were considered recovered when they were symptom-free at rest according to clinical interview and PCSS score, had a normal neurological examination, were tolerating full school activities and exercise without symptoms, and completed the graduated Return-to-Play protocol set forth by the Concussion In Sport Group consensus statement [1]. SRF patients were considered clinically recovered when satisfactory healing of the fracture was confirmed by post-injury radiography and clinical examination and the patient was cleared to return to full physical and school activities without restriction.

### Data analysis

A 5% drop in grades (e.g., a reduction from 85% to 80%) was considered a minimally clinically important difference. Assuming a 1:1 ratio between SRC and SRF, a standard deviation of 8%, power of 80%, and two-sided alpha of 0.05, a total of 82 youth (41 SRC and 41 SRF) were required.

Dichotomous and polychotomous characteristics and outcomes for students with a SRC versus SRF were summarized as percentages and compared using the χ2 test. Normally distributed continuous characteristics were summarized as means with standard deviations (or 95% confidence intervals) and compared using an independent t-test. Non-normally distributed characteristics were summarized as medians with interquartile ranges and compared using a rank sum test. For all analyses, a two-sided p-value less than 0.05 was interpreted as statistically significant. For each student, a change in overall grades from pre-injury to post-injury was calculated. The mean change in grades with 95% confidence intervals was determined for students with a SRC or SRF. The mean difference between the two injury types was calculated.

Linear regression with manual purposeful backward elimination was performed to determine post-injury grades while adjusting for pre-injury grades. Effect modification by age, sex, history of previous concussion, previous headaches or migraines, and private versus public school was examined. Next, age, sex, history of previous concussion, and private versus public school were examined as potential confounders. The potential confounder that had the smallest effect on the effect estimate was removed from the model. The process was stopped once the remaining confounder(s) changed the effect estimate by more than 10% [15]. This was repeated for post-injury core grades as the outcome while adjusting for pre-injury core grades. Change in overall and core grades were examined for the following subgroups: age, sex, history of previous concussion, previous headaches or migraines, and private versus public school attendance. Sensitivity analysis were conducted using difference in differences regression techniques and with post injury grades as the outcome after controlling for pre-injury grades. Negative binomial regression using backwards elimination techniques was used to calculate the incident rate ratio (with 95% confidence intervals) for missed school for students with a SRC or SRF. Effect modification by age and sex were examined followed by the examination of the abovementioned potential confounders.

The accommodations were tabulated and reported as most helpful, somewhat helpful, not helpful, not available, and not applicable and compared using the χ2 test by injury type.

### Ethical approval

Ethical approval was granted by the Bannatyne Health Research Ethics Board at the University of Manitoba.

## Results

In total, 250 students were recruited; however, 19 students were excluded because they attended only their first appointment. Of the 231 students, an additional 27 were lost to follow-up and one student sustained a second injury while recovering from their first and was subsequently excluded. Thus, there were 203 students included in the study who completed school attendance data. The median time from injury to initial assessment was 6 days (IQR: 5, 10) for SRC students and 9 days (IQR: 7, 12) for SRF students (p = 0.090). The median time to physician-documented clinical recovery was 26 days (IQR: 17, 43) for SRC students and 31 days (IQR: 23, 42) for SRF students (p = 0.128).

### Baseline characteristics

There were no significant differences among those with a SRC or SRF with the exception that students who sustained a concussion were significantly older and were more likely to have sustained a previous concussion (Table 1). With the exception of those who played hockey were significantly more likely be included, there were no significant differences in the baseline characteristics of those who were recruited versus those who were included and completed school attendance data.

**Table 1:** Baseline characteristics of students with complete school attendance data who presented with a sport-related concussion or a sport-related fracture.

	Total N = 203	Sport-related concussion N = 126	Sport-related fracture N = 77	p-value
Age, mean (SD)	14.5 (1.2)	14.8 (1.3)	14.1 (1.0)	<0.0001
Missing		2	1	
Male (%)	123 (60.6)	76 (60.3)	47 (61.4)	0.919
History of ADHD (%)	9 (4.3)	5 (4.0)	4 (5.2)	0.680
History of depression	8 (3.9)	6 (4.8)	2 (2.6)	0.442
History of non-specific or migraine headache (%)	23 (11.3)	18 (14.3)	5 (6.5)	0.089
History of previous concussion (%)	84 (41.4)	74 (58.7)	10 (12.3)	<0.0001
Attending a private school (%)	31 (15.3)	21 (16.7)	10 (13.0)	0.479
Sport played at the time of injury				
Hockey (%)	80 (39.4)	64 (50.8)	16 (20.785)	
Football (%)	32 (15.8)	17 (13.5)	15 (19.5)	
*Soccer (%)	28 (13.8)	17 (8.4)	11 (14.3)	
Basketball (%)	22 (10.5)	8 (6.3)	14 (18.2)	
Other (%)	42 (20.7)	21 (16.7)	21 (27.3)	
Initial PCSS (median IQR)	17 (6, 39)			

*1 patient reported a head injury while playing soccer and hockey in the same day

### Overall and core grades

Of the 203 students, seven students were injured and recovered during the summer holidays and not included in the grades analysis and 124 (63.3%) students submitted both pre- and post-injury report cards. There was no significant difference in sex (p = 0.507), age (p = 0.621), history of depression (p = 0.755), history of ADHD (p = 0.922), history of migraines (p = 0.609), or attending private school (p = 0.367) among those who did and did not submit both report cards. Students with a history of concussion were significantly less likely to submit both report cards (p = 0.044). Students who suffered a SRC were significantly older and more likely to have sustained a previous concussion than those with a SRF. There were no other differences in baseline characteristics (Table 1).

There was no significant differences in change (pre-injury to post-injury) overall grades or core grades among students who suffered a SRC or SRF (Table 2). While males with SRF exhibited a significant decline in overall grades, their decline was not significantly different than those with a SRC. Similarly, those with a SRF who attended private school also exhibited a significant decline in change in core grades; however their decline was not significantly greater than those with a SRC. There were no significant differences in change in overall or core grades between the two groups when stratified by sex, age, history of previous concussion, or public versus private school attendance. While those who were placed on a RTL program experienced a decline in grades compared to those who were not placed on an RTL grogram, the change in overall grades (p = 0.052) or core grades (p = 0.110) was not statistically significant.

**Table 2:** Change in overall and core grades for students with a sport-related concussion versus sport-related fracture (% change with 95% CI).

	Change in Overall Grades (Pre-Post)			Change in Core Grades (Pre-Post)		
	Sport-related concussion	Sport-related fracture	Difference between groups	Sport-related concussion*	Sport-related fracture	Difference between groups
All Students	N = 92 -1.0 (-2.1, 0.1)	N = 32 -0.9 (-2.1, 0.3)	0.1 (-1.9, 2.0)	N = 91 -0.9 (-2.3, 0.6)	N = 32 -0.7 (-1.9, 0.6)	0.2 (-2.4, 2.8)
Males	N = 58 -1.4 (-3.0, 0.2)	N = 15 **-1.7** **(-3.2, -0.3)**	-0.3 (-3.5, 2.9)	N = 58 -1.0 (-3.1, 1.2)	N = 15 -0.9 (-3.4, 1.6)	0.1 (-4.3, 4.4)
Females	N = 34 -0.2 (-1.4, 1.0)	N = 17 -0.2 (-2.1, 1.7)	0.0 (-2.1, 2.1)	N = 33 -0.7 (-2.4, 1.1)	N = 17 -0.4 (-1.8, 1.0)	0.3 (-2.3, 2.8)
13–14 years old	N = 44 -1.0 (-2.2, 0.2)	N = 24 -0.3 (-1.5, 1.0)	0.7 (-1.1, 2.5)	N = 43 -1.0 (-2.5, 0.5)	N = 24 -0.3 (-1.5, 1.0)	0.71 (-1.5, 2.9)
15–17 years old	N = 46 -1.4 (-3.2, 0.5)	N = 8 -2.8 (-5.8, 0.2)	-1.4 (-5.9, 3.0)	N = 46 -1.5 (-3.7, 0.8)	N = 8 -1.8 (-6.1, 2.5)	-0.3 (-5.9, 5.3)
History of previous concussion	N = 54 -1.3 (-3.0, 0.4)	N = 5 -2.1 (-4.6, 0.3)	-0.8 (-6.5, 4.9)	N = 54 -1.2 (-3.5, 1.1)	N = 5 -3.3 (-7.2, 0.6)	-2.1 (-9.6, 5.4)
No history of previous concussion	N = 38 -0.5 (-1.6, 0.6)	N = 27 -0.7 (-2.0, 0.7)	-0.2 (-1.9, 1.5)	N = 37 -0.4 (-2.1, 1.3)	N = 27 -0.2 (-1.5, 1.2)	0.2 (-2.0, 2.5)
Attending a public school	N = 75 -1.2 (-2.5, 0.1)	N = 26 -0.6 (-1.9, 0.7)	0.6 (-1.7, 2.9)	N = 26 -0.5 (-2.1, 1.1)	N = 74 -1.4 (-3.1, 0.3)	0.9 (-2.1, 3.9)
Attending a private school	N = 17 -0.1 (-1.7, 1.6)	N = 6 2.4 (-5.9, 1.2)	-2.3 (-5.5, 0.9)	N = 17 1.4 (-1.6, 4.4)	**N = 6** **-1.4** **(-2.6, -0.1)**	-2.8 (-7.8, 2.3)

*One concussion student was not enrolled in any core classes

### Overall grades

Accounting for pre-injury grades, there was no significant difference in post-injury grades among students with a SRC versus SRF (-0.1%; 95% CI: -2.1, 1.8). There was no evidence of effect modification by age (p = 0.21), sex (p = 0.97), history of previous concussion (p = 0.73), or attending public or private school (p = 0.26). After adjusting for pre-injury grades, age, sex, and attending public or private school, there was no significant difference in post-injury grades among students with a SRC versus SRF (0.7%; 95% CI: -1.5, 2.9). While only sex and history of previous concussion confounded the relationship between post-injury grades and injury type, the association was not statistically significant when accounting for pre-injury grades (0.7; 95% CI: -1.5, 2.8). There was no difference in the results when derived using differences in differences regression or determining post-injury grades after controlling for pre-injury school performance.

### Core grades

After controlling for pre-injury core subject grades, there was no significant difference in post-injury core subject grades among students with a SRC versus SRF (-0.4%; 95% CI: -3.0, 2.2). There was no evidence of effect modification by age (p = 0.24), sex (p = 0.99), history of previous concussion, p = 0.61), or attending public or private school (p = 0.35). After adjusting for pre-injury grades, age, sex, and attending public or private school, there was no significant difference in post-injury grades among students with a SRC versus SRF (-0.1%; 95% CI: -2.9, 2.6). Although the relationship between post-injury core grades and injury type was confounded by sex and previous concussion, the association was not statistically significant when accounting for pre-injury grades (0.5; 95% CI: -2.3, 3.4). The results were similar when examined using differences in differences regression or determining post-injury grades after controlling for pre-injury school performance.

### School attendance

Of the 231 potential students who attended at least two appointments, 203 (87.9%) students returned their school attendance log books.

Students who sustained a SRC missed significantly more school (median 4 full or partial days of school [IQR: 1, 7] compared with students who sustained a SRF (median 1 full or partial day [IQR: 0, 4], p<0.0001). This was evident in the crude rate ratio demonstrating that SRC students missed significantly more school than students with a SRF (IRR: 2.53; 95% CI: 1.72, 3.72).

There was no evidence of effect modification by age (p = 0.281) or sex (p = 0.127). After controlling for age, sex, history of depression, private versus public school attendance, history of non-specific or migraine headaches, and history of previous concussion, students with a SRC had twice the risk of missing school compared to those with a SRF (IRR: 2.09; 95% CI: 1.30, 3.36). Of the abovementioned variables, only sex confounded the relationship (IRR: 2.32; 95% CI: 1.59, 3.41). Sex was also significantly associated with missing school where boys missed significantly less school than girls (IRR: 0.56; 95% CI: 0.39, 0.82), regardless of their type of injury.

### School accommodations

Overall, 190 (76.0%) students completed the exit interview assessing school accommodations (SRC: 113; SRF: 77). Students with a SRC found reduced attendance, not participating in physical education class, and open communication with teachers to be the most helpful accommodations (Table 3). Students with a SRF reported not participating in physical education class, extra time to complete exams and taking breaks as needed to be the most helpful. Overall, 60 (53.1%) students with SRC rated their school as very accommodating while 31 (40.3%) students with SRF rated their school as very accommodating but this was not statistically significant (p = 0.082). In total, 56 (49.6%) of SRC students received a Return to Learn program.

**Table 3:** Perceived helpfulness of academic accommodations.

		Sport-related concussion N = 113 (%)	Sport-related fracture N = 77 (%)
Reduced attendance	Not helpful	1 (0.9)	2 (2.6)
	Somewhat helpful	8 (7.1)	16 (20.8)
	Very helpful	71 (62.8)	12 (15.6)
	Not applicable	28 (24.8)	38 (49.4)
	Not available	5 (4.4)	6 (7.8)
Reduced workload	Not helpful	4 (3.5)	7 (9.1)
	Somewhat helpful	18 (15.9)	8 (10.4)
	Very helpful	44 (38.9)	7 (9.1)
	Not applicable	33 (29.2)	40 (52.0)
	Not available	14 (12.9)	12 (15.6)
Extra time for assignments or tests	Not helpful	5 (4.4)	10 (13.0)
	Somewhat helpful	19 (16.8)	5 (6.5)
	Very helpful	50 (44.3)	14 (18.2)
	Not applicable	33 (29.2)	33 (42.9)
	Not available	6 (5.3)	12 (15.6)
Breaks from class	Not helpful	2 (1.8)	7 (9.1)
	Somewhat helpful	25 (22.1)	6 (7.8)
	Very helpful	48 (42.5)	14 (18.2)
	Not applicable	29 (25.7)	36 (46.8)
	Not available	9 (8.0)	11 (14.3)
Reschedule or excused from exams	Not helpful	3 (2.7)	6 (7.8)
	Somewhat helpful	13 (11.5)	7 (9.1)
	Very helpful	55 (48.7)	8 (10.4)
	Not applicable	36 (31.9)	42 (54.6)
	Not available	6 (5.3)	11 (14.3)
Quiet workplace	Not helpful	5 (4.4)	11 (14.3)
	Somewhat helpful	21 (18.6)	4 (5.2)
	Very helpful	26 (23.0)	4 (5.2)
	Not applicable	46 (40.7)	47 (61.0)
	Not available	15 (13.3)	7 (9.1)
Excused from taking notes	Not helpful	8 (7.1)	12 (15.6)
	Somewhat helpful	14 (12.4)	6 (7.8)
	Very helpful	27 (23.9)	9 (11.7)
	Not applicable	51 (45.1)	38 (49.4)
	Not available	13 (11.5)	9 (11.7)
Reduced computer use	Not helpful	8 (7.1)	15 (19.5)
	Somewhat helpful	9 (8.0)	4 (5.2)
	Very helpful	33 (29.2)	6 (7.8)
	Not applicable	52 (46.0)	43 (55.8)
	Not available	11 (9.7)	6 (7.8)
Avoid noisy environments	Not helpful	11 (9.7)	12 (15.6)
	Somewhat helpful	15 (13.3)	5 (6.5)
	Very helpful	11 (9.7)	4 (5.2)
	Not applicable	56 (49.6)	47 (61.0)
	Not available	20 (17.7)	5 (6.5)
Excused from physical education class	Not helpful	1 (0.9)	8 (10.4)
	Somewhat helpful	13 (11.5)	15 (19.5)
	Very helpful	60 (53.1)	30 (39.0)
	Not applicable	37 (32.7)	19 (24.7)
	Not available	2 (1.8)	2 (2.6)
Communication with teachers	Not helpful	10 (8.9)	5 (6.5)
	Somewhat helpful	28 (24.8)	19 (24.7)
	Very helpful	62 (54.9)	29 (37.7)
	Not applicable	9 (8.0)	16 (20.8)
	Not available	4 (3.5)	5 (6.5)
Overall school was accommodating	Not accommodating	2 (1.8)	6 (7.8)
	Somewhat accommodating	46 (40.7)	30 (39.0)
	Very accommodating	60 (53.1)	31 (40.3)
	Missing	5 (4.4)	4 (5.2)

## Discussion

This study provides novel insight into the effect of SRC on objective academic outcomes including school grades and attendance and highlights specific academic accommodations that can benefit students recovering from this injury.

First, we found no significant change in overall or core school grades among students who had sustained a SRC or SRF. These results align with previous research examining objective academic outcomes after concussion. A recent systematic review found minimal effects on academic performance (change in grade-point average or national exam scores) after sustaining a concussion or mild traumatic injury compared with non-injured students or students with a non-head brain injury [4]. A population-based controlled study of grade 9–12 students found no significant difference in the overall change in pre- and post-injury grades between students with a without a concussion and observed no difference in graduation rates between these two groups [16]. The results of the present study are also in agreement with a more recent study that found no significant difference in pre- and post-injury grades in adolescent SRC patients even after controlling for important modifiers of concussion recovery including the development of persistent post-concussion symptoms [5]. In the present study, we did find that male students with a SRF did have a significant decrease in their overall grades (-1.73%); however, this decrease was not significantly different than males who sustained a SRC.

Second, we found that students who sustained a SRC missed four times as many days of school compared to those who sustained a SRF. This is consistent with the results of previous studies that have found that students diagnosed with a concussion typically miss 2–5 days of school [8,17–19]. The results are also consistent with a previous study that found that students with mild TBI missed more days of school than those with an orthopedic extremity injury [20]. Given that concussion symptoms such as headaches, photosensitivity, trouble focusing, and difficulties with memory and concentration can be exacerbated by school activities and environments, these results are not surprising and point to the important need for some students to take limited time off of school before returning following this unique injury.

Lastly, this study provides important insight into the specific academic accommodations students recovering from a SRC find most helpful in allowing a gradual return to school activities and that should be emphasized in Return-to-Learn programs. In this study, the academic accommodations that students felt were most helpful during recovery from their SRC were variable and included those such as reduced attendance, not participating in physical education class, and open communication with teachers. As suggested by previous authors [7,21–23], individually tailored Return-to-Learn programs can be an important resource to help facilitate communication between all stakeholders involved in a student-athlete’s recovery and ensure that students are receiving specific academic accommodations based on the nature and severity of concussion symptoms and the predicted length of clinical recovery. Although concussion has been viewed in the past as an “invisible injury”, it is encouraging that 94% students with a SRC reported feeling their school was somewhat or very accommodating compared to the 79% of students with SRF who returned to school with a more visible injury.

### Limitations

There are several limitations associated with this study. First, the academic outcomes analysis was impacted by the challenge of obtaining both pre-injury and post-injury report cards from the SRF students and parents, despite multiple attempts to contact study participants and obtain these. Those with a previous concussion were significantly less likely to submit both report cards and this may have introduced a selection bias into our findings. Failure to obtain report cards resulted in a reduction in the sample size used to examine the effects of SRC and SRF on school grades. However, this is a well-acknowledged challenge among studies examining academic outcomes in concussion and mild TBI and is difficult to overcome [24–26]. Despite this limitation, the mean change in grades between SRC and SRF student included in this study (<1%) is unlikely to be clinically meaningful or impact the student’s long term academic trajectory (i.e., graduation status, acceptance into post-secondary programs). Similarly, there were two subgroup analyses of change in grades that were statistically significant; however they should be interpreted cautiously as several subgroup analyses were performed and there is the potential for significant results occurring due to chance. Second, the variability in the timing of injury within school term and the number of terms each school has may have had an impact on school grades. Students who were injured at the beginning of the school term would have had additional time to recover from their injuries and make up for missed school compared to those who were injured closer to the end of the term and the results may have been confounded by this. However, on average, for a selection bias to effect the results, the SRC students would have to be injured at the beginning of the term and SRF students at the end of the term of vice versa and this is unlikely. Therefore, we do not expect that timing of the injury would have varied by injury type and biased our results. Third, it was not possible to ensure that students had the same number of exams or assignments included within each reporting term. Similarly, school divisions, public versus private schools and individual teachers at the same school may use different standards when assigning grades. Again, the number of assignments or exams and grading criteria should not have varied by type of injury. Although it is a rare occurrence, some students may have enrolled in a different school midway through their recovery and this may bias the results if this occurred more frequently in one of the two injury groups. Fourth, it is possible that certain variables that impact school performance following these injuries were not collected. For example, hand dominance was not collected among students who sustained an SRF and extra time spent studying after the injury were not collected. Students who injured their dominant upper extremity may have had greater difficulty returning to school compared to those who injured a lower extremity or non-dominant upper extremity. Other factors such as family functioning and socioeconomic status may also influence academic performance and were also not collected. Our previous population-based study did suggest an important modifying effect of socioeconomic status on school assigned grades following concussion and this variable should be considered in future studies. Fifth, a proportion of the students with SRC were provided with individually-tailored Return-to-Learn Programs, which may have impacted their school grades, which academic accommodations they received, and their perceptions of how accommodating they felt their school was following their injury. Nonetheless, it remains unknown whether teachers received the Return-to-Learn programs or incorporated the outlined recommendations. A sensitivity analysis revealed that the change in grades was similar among the SRC students who did or did not receive an RTL program. It is possible that post-injury instructions provided by referring physicians may have had an impact on the number of days a child missed from school. It is also possible that report cards are distributed too infrequently to be impacted by cognitive deficits that can occur after a SRC. However, if there are short-term academic deficits after a SRC, they appear to be resolved by the end of the school term. Additional work is required to obtain a more sensitive measure of school performance, such as individual assignments, quiz or test scores, or teacher evaluations. Lastly, children and adolescents with orthopaedic injuries have also been found to experience concussion-like symptoms that may impact school performance and participation [27]. Concussion symptoms were not assessed among SRF patients in this study and this should be considered in future studies.

### Conclusion and future directions

This study is the first to demonstrate that students who sustain a SRC miss significantly more days of school but demonstrate similar changes in school report-card grades post-injury compared to those with a SRF. Physicians should be aware that SRC is associated with symptoms that can present a challenge for student-athletes returning to school. Future research is needed to identify patient risk factors for developing difficulty in school and how to best engage schools in following RTL programs or ensure students are given the school-related accommodations they needed following a concussion.
